# CCR2 Regulates the Uptake of Bone Marrow-Derived Fibroblasts in Renal Fibrosis

**DOI:** 10.1371/journal.pone.0077493

**Published:** 2013-10-10

**Authors:** Yunfeng Xia, Mark L. Entman, Yanlin Wang

**Affiliations:** 1 Division of Nephrology, Department of Medicine, Baylor College of Medicine, Houston, Texas, United States of America; 2 Division of Nephrology, Department of Medicine, Guangdong General Hospital, Guangdong Academy of Medical Science, Guangzhou, China; 3 Division of Cardiovascular Sciences and the DeBakey Heart Center, Department of Medicine, Baylor College of Medicine and the Methodist Hospital, Houston, Texas, United States of America; UCL Institute of Child Health, United Kingdom

## Abstract

Recent studies have shown that bone marrow-derived fibroblasts contribute significantly to the pathogenesis of renal fibrosis. However, the molecular mechanisms underlying the recruitment of bone marrow-derived fibroblasts into the kidney are incompletely understood. Bone marrow-derived fibroblasts express the chemokine receptor - CCR2. In this study, we tested the hypothesis that CCR2 participates in the recruitment of fibroblasts into the kidney during the development of renal fibrosis. Bone marrow-derived collagen-expressing GFP^+^ fibroblasts were detected in the obstructed kidneys of chimeric mice transplanted with donor bone marrow from collagen α1(I)-GFP reporter mice. These bone marrow-derived fibroblasts expressed PDGFR-β and CCR2. CCR2 knockout mice accumulated significantly fewer bone marrow-derived fibroblast precursors expressing the hematopoietic marker-CD45 and the mesenchymal markers-PDGFR-β or procollagen I in the obstructed kidneys compared with wild-type mice. Furthermore, CCR2 knockout mice displayed fewer bone marrow-derived myofibroblasts and expressed less α-SMA or FSP-1 in the obstructed kidneys compared with wild-type mice. Consistent with these findings, genetic deletion of CCR2 inhibited total collagen deposition and suppressed expression of collagen I and fibronectin. Moreover, genetic deletion of CCR2 inhibits MCP-1 and CXCL16 gene expression associated with a reduction of inflammatory cytokine expression and macrophage infiltration, suggesting a linear interaction between two chemokines/ligand receptors in tubular epithelial cells. Taken together, our results demonstrate that CCR2 signaling plays an important role in the pathogenesis of renal fibrosis through regulation of bone marrow-derived fibroblasts. These data suggest that inhibition of CCR2 signaling could constitute a novel therapeutic approach for fibrotic kidney disease.

## Introduction

Renal fibrosis is the final common manifestation of chronic kidney disease [1,2]. Furthermore, tubulointerstitial fibrosis is a key structural component of obstructive nephropathy, which is the major cause of chronic kidney disease in children [3]. Renal interstitial fibrosis is characterized by fibroblast activation and excessive production and deposition of extracellular matrix (ECM), which results in the destruction of renal parenchyma and progressive loss of kidney function. Because activated fibroblasts are the principal cells responsible for ECM production, their activation is regarded as a key event in the pathogenesis of renal fibrosis [4-6]. However, the origin of these fibroblasts remains unsettled. They are traditionally thought to arise from resident renal fibroblasts. Recent evidence indicates that they may originate from bone marrow-derived fibroblast progenitor cells [7-11]. 

Bone marrow-derived fibroblast precursors termed fibrocytes are derived from a subpopulation of monocytes via monocyte-to-fibroblast transition [12-16]. These cells express mesenchymal markers such as collagen I and vimentin and hematopoietic markers such as CD45 and CD11b [12,17-19]. These cells in culture display an adherent, spindle-shape morphology and express α-SMA that is enhanced in response to TGF-β1, consistent with the notion that they can differentiate into myofibroblasts [17-19]. Recent studies provide strong evidence that bone marrow-derived fibroblast precursors migrate into the kidney and contribute significantly to the development of renal fibrosis [8-11,20]. However, the molecular mechanisms underlying the recruitment of these cells into injured kidneys are not fully understood. 

Chemokines play primary roles in mediating the trafficking of circulating cells to sites of injury via activation of their seven-transmembrane G protein-coupled receptors [21]. Since bone marrow-derived fibroblasts express the chemokine receptor CCR2 [22], we investigated the role of CCR2 in renal fibrosis using CCR2 knockout (KO) mice. Our results showed that CCR2 deficiency inhibited renal fibrosis through suppression of myeloid fibroblast infiltration into the kidney.

## Materials and Methods

### Animals

Animal experiments were approved by the Institutional Animal Care and Use Committee of Baylor College of Medicine (IACUC permit #: AN-5011). The investigation conforms with the recommendations in the Guide for the Care and Use of Laboratory Animals published by the US National Institutes of Health (NIH Publication No. 85-23, revised 1996). All efforts were made to minimize suffering. The CCR2 KO mice on a background of C57BL/6J were purchased from the Jackson Laboratory. Transgenic mice, expressing GFP driven by collagen α1(I) promoter, were a generous gift from Dr. David A. Brenner[23]. Male WT or CCR2 KO mice (*8-12 weeks old*, weighing 20–30 g) were anesthetized by i.p. injection of ketamine (80 mg/kg) and xylazine (10 mg/kg). Through a flank incision, the left ureter was exposed and completely ligated using fine suture material (4–0 silk) at two points [10,24]. Mice were allowed to recover from anesthesia and were housed in standard rodent cages with *ad libitum* access to water and food until sacrificed.

### Bone Marrow Transplantation

 Bone marrow transplantation was performed as described previously [10]. Briefly, bone marrow cells (5X10^6^) from CoI1A1-GFP mice were transferred to lethally irradiated C57BL/6 mice. Chimeric mice were allowed to recuperate for 2 months prior to induction of kidney injury by UUO. 

### Renal Morphology

Mice were euthanized and perfused by injections of PBS into the left ventricle of the heart to remove blood. One portion of the kidney tissue was fixed in 10% buffered formalin and embedded in paraffin, cut at 4 µm thickness, and stained with picrosirius red to identify collagen fibers. The picrosirius red-stained sections were scanned using a microscope equipped with a digital camera (Nikon, Melville, NY), and quantitative evaluation was performed using NIS-Elements Br 3.0 software. The collagen-stained area was calculated as a percentage of the total area.

### Quantitative Real-Time RT-PCR

 Quantitative analysis of the target mRNA expression was performed with real-time reverse transcription – polymerase chain reaction (RT-PCR) by the relative standard curve method [10]. Total RNA was extracted from snap-frozen kidney tissues with TRIzol Reagent (Invitrogen). Total RNA was reverse-transcribed and amplified in triplicate using IQ SYBR green supermix reagent (Bio-Rad, Herculus, CA) with a real-time PCR machine (Bio-Rad, Herculus, CA), according to the manufacturer’s instructions. The specificity of real-time PCR was confirmed by melting-curve analysis. The expression levels of the target genes were normalized to the GAPDH level in each sample. The following are the primer sequences: Monocyte chemoattractant protein 1 (MCP-1): Forward 5′-TCACCTGCTGCTACTCATTCACCA-3′ and reverse 5′- TACAGCTTCTTTGGGACACCTGCT-3′; CXCL16: Forward 5′- ACCCTTGTCTCTTGCGTTCTTCCT-3′ and reverse 5′- ATGTGATCCAAAGTACCCTGCGGT-3′; TNF-α: Forward 5′- CATGAGCACAGAAAGCATGATCCG-3′ and reverse 5′- AAGCAGGAATGAGAAGAGGCTGAG-3′; IFN-γ: Forward 5′- CTTCAGCAACAGCAAGGCGAAA-3′ and reverse 5′- ATCAGCAGCGACTCCTTTTCCG-3′; GAPDH: Forward 5′-TGCTGAGTATGTCGTGGAGTCTA-3′ and reverse 5′-AGTGGGAGTTGCTGTTGAAATC-3′.

### Immunofluorescence

Renal tissues were embedded in OCT compound, snap-frozen on dry ice, cut at 5 µm thickness, and mounted on microscope slides. After fixation, nonspecific binding was blocked with serum-free protein block (DAKO). Slides were then incubated with goat anti-MCP-1 antibody (R&D Systems) followed by Alexa-488 conjugated donkey anti-goat antibody (Invitrogen), rabbit anti-collagen I antibody (Rockland) followed by Alexa-488 conjugated donkey anti-rabbit antibody (Invitrogen), rabbit anti-fibronectin antibody (Sigma) followed by Alexa-488 conjugated donkey anti-rabbit antibody (Invitrogen), or rabbit anti-α-SMA antibody (Abcam) followed by Alexa-488 conjugated donkey anti-rabbit antibody (Invitrogen). For double immunofluorescence, kidney sections were fixed and stained with primary antibodies followed by appropriate secondary antibodies sequentially. Slides were mounted with mounting medium containing DAPI. Fluorescence intensity was visualized using a microscope equipped with a digital camera (Nikon, Melville, NY). Quantitative evaluation of sections stained with antibodies to α-SMA, collagen I and fibronectin was performed using NIS-Elements Br 3.0 software. The fluorescence positive area was calculated as a percentage of the total area.

### Western Blot Analysis

Protein was extracted using the RIPA buffer containing cocktail proteinase inhibitors (Thermo Fisher Scientific Inc., Rockford, IL) and quantified with Bio-Rad protein assay. Equal amounts of protein were separated on SDS–polyacrylamide gels in a Tris/glycine buffer system, transferred onto nitrocellulose membranes, and blotted according to standard procedures with primary antibodies (collagen I, fibronectin, and α-SMA). Membranes were then stripped and reblotted with anti-GAPDH antibody (Millipore, Billerica, CA). The specific bands of target proteins were analyzed using an Odyssey IR scanner and band intensities were quantified using NIH Image/J.

### Tubular Epithelial Cell Culture

 The mouse kidney tubular epithelial cell line (TCMK-1, CCL-139) was obtained from the American Type Culture Collection and maintained in Dulbecco's modified Eagle's medium (DMEM) containing 10% fetal bovine serum (FBS) and 1% penicillin and streptomycin in a humidified 5% CO2/95% air incubator at 37°C. Cells were made quiescent by starvation in DMEM with 1% FBS overnight and then were treated with TNF-α (10 ng/ml) and IFN-γ (10ng/ml) for 24 hr.

### Statistical Analysis

All data were expressed as mean ± SEM. Kolmogorov-Smirnov test was performed to ascertain the normal distribution of the data. Multiple group comparisons were performed by one-way ANOVA followed by the Bonferroni procedure for comparison of means. *P* < 0.05 was considered statistically significant.

## Results

### Bone Marrow-derived Fibroblasts Express CCR2

**Figure 1:**
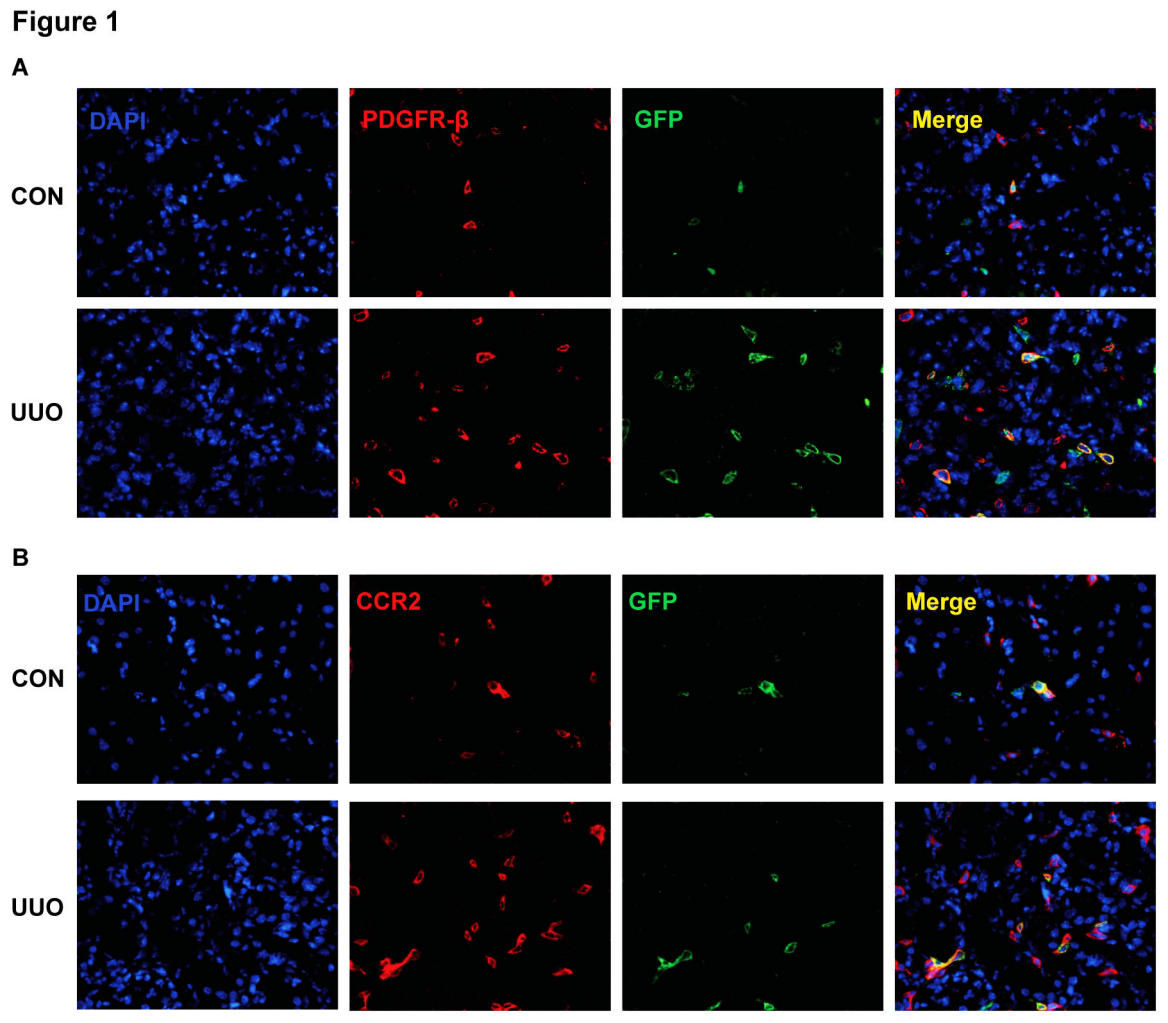
Bone marrow-derived fibroblasts express PDGFR-β and CCR2. **A**. Representative photomicrographs of kidney sections stained for PDGFR-β (red) and counter stained with DAPI (blue) (Original magnification: X400). **B**. Representative photomicrographs of kidney sections stained for CCR2 (red) and counter stained with DAPI (blue) (Original magnification: X400).

We have shown that bone marrow-derived fibroblasts migrated into the kidney in response to UUO [10,16]. To confirm the bone marrow origin of these fibroblasts, we generated chimeric mice that express GFP driven by collagen α1(I) promoter. Two months after bone marrow transplantation, chimeric mice were subjected to UUO for 7 days. Kidney sections were stained for platelet-derived growth factor receptor β (PDGFR-β), a mesenchymal marker, and examined with a fluorescence microscope. Our results showed that GFP and PDGFR-β dual positive cells were detected abundantly in the obstructed kidneys, but rarely seen in the contralateral kidneys (Figure 1A). GFP^+^ cells accounted for 35-40% of PDGFR-β^+^ cells. These data indicate that bone marrow-derived fibroblasts express PDGFR-β. 

To examine if recruited bone marrow-derived fibroblasts in the kidney express CCR2, kidney sections were stained for CCR2 and examined with a fluorescence microscope. Our results demonstrated that GFP and CCR2 dual positive cells are detected abundantly in the obstructed kidneys, but rarely seen in the contralateral kidneys (Figure 1B). These results indicate that bone marrow-derived fibroblasts recruited into the kidney express CCR2.

### CCR2 Deficiency Impairs Myeloid Fibroblasts Accumulation

**Figure 2:**
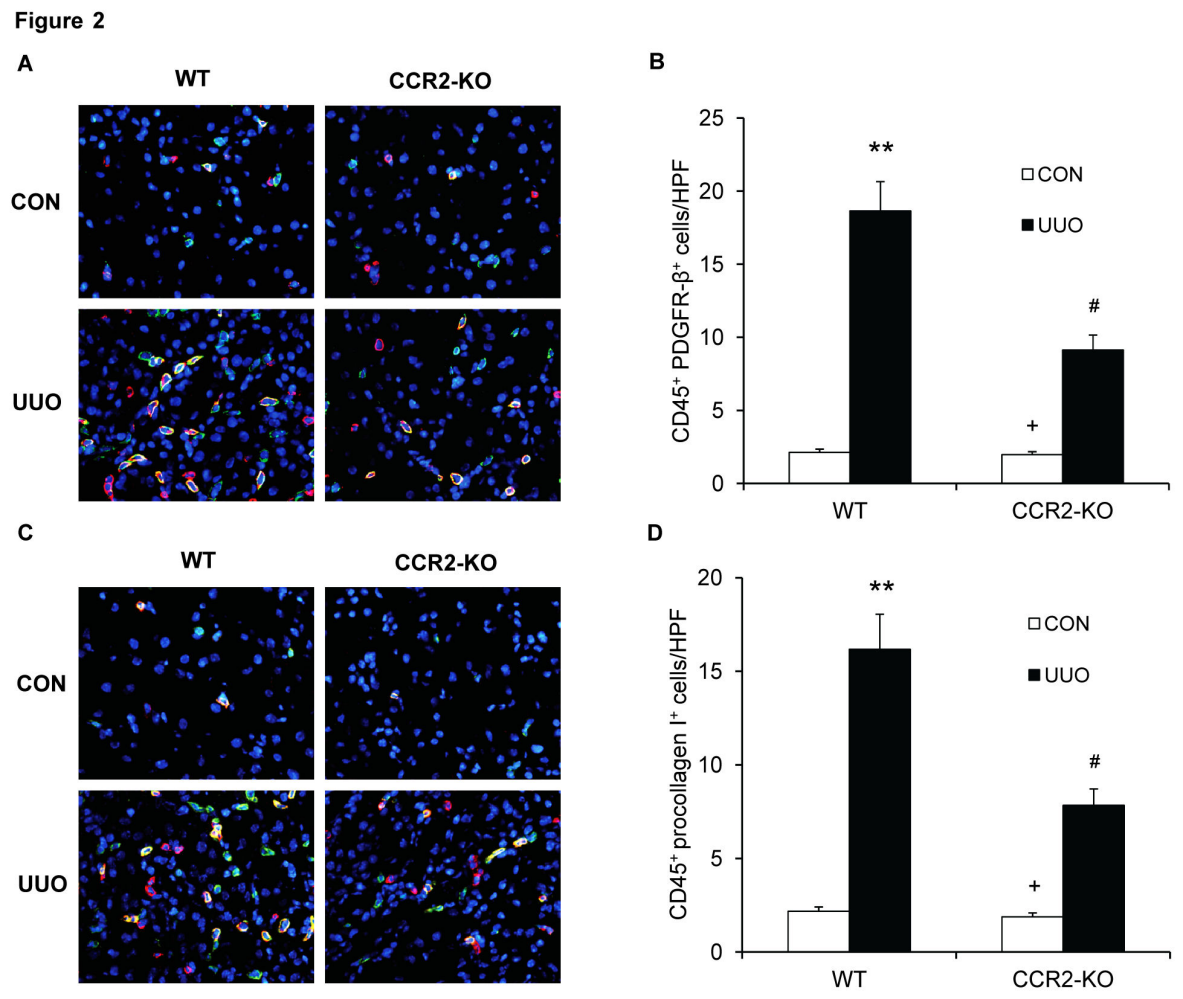
CCR2 deficiency suppresses the accumulation of bone marrow-derived fibroblasts in the kidneys after UUO. **A**. Representative photomicrographs of kidney sections from WT and CCR2-KO mice 1 week after UUO stained for CD45 (red), PDGFR-β (green), and DAPI (blue). **B**. Quantitative analysis of CD45^+^ and PDGFR-β^+^ fibroblasts in the kidneys of WT and CCR2-KO mice 1 week after UUO. ** *P* < 0.01 vs WT controls, ^#^ *P* < 0.05 vs WT-UUO, and ^+^ *P* < 0.05 vs KO-UUO. n=6 per group. **C**. Representative photomicrographs of kidney sections from WT and CCR2-KO mice 1 week after UUO stained for CD45 (red), procollagen I (green), and DAPI (blue). **D**. Quantitative analysis of CD45^+^ and procollagen I^+^ fibroblasts in the kidneys of WT and CCR2-KO mice 1 week after UUO. ** *P* < 0.01 vs WT controls, ^#^ *P* < 0.05 vs WT-UUO, and ^+^ *P* < 0.05 vs KO-UUO. n=6 per group.

 To examine if CCR2 plays a role in the accumulation of bone marrow-derived fibroblasts in the kidneys, WT and CCR2-KO mice were subjected to obstructive injury for 7 days. Kidney sections were stained for CD45 and PDGFR-β or CD45 and procollagen I and examined with a fluorescence microscope. Our results showed that the number of CD45^+^ and PDGFR-β^+^ fibroblasts or CD45^+^ and procollagen I^+^ fibroblasts was markedly increased in obstructed kidneys of WT mice. In contrast, the number of CD45^+^ and PDGFR-β^+^ fibroblasts or CD45^+^ and procollagen I^+^ fibroblasts was significantly reduced in obstructed kidneys of CCR2-KO mice (Figure 2). Similar results were obtained for after WT and CCR2-KO were subjected to obstructive injury for 5 days (Figure S1). These data indicate that CCR2 signaling plays an important role in recruiting bone marrow-derived fibroblasts into the kidney.

### CCR2 Deficiency Reduces MCP-1 and CXCL16 Gene Expression

**Figure 3:**
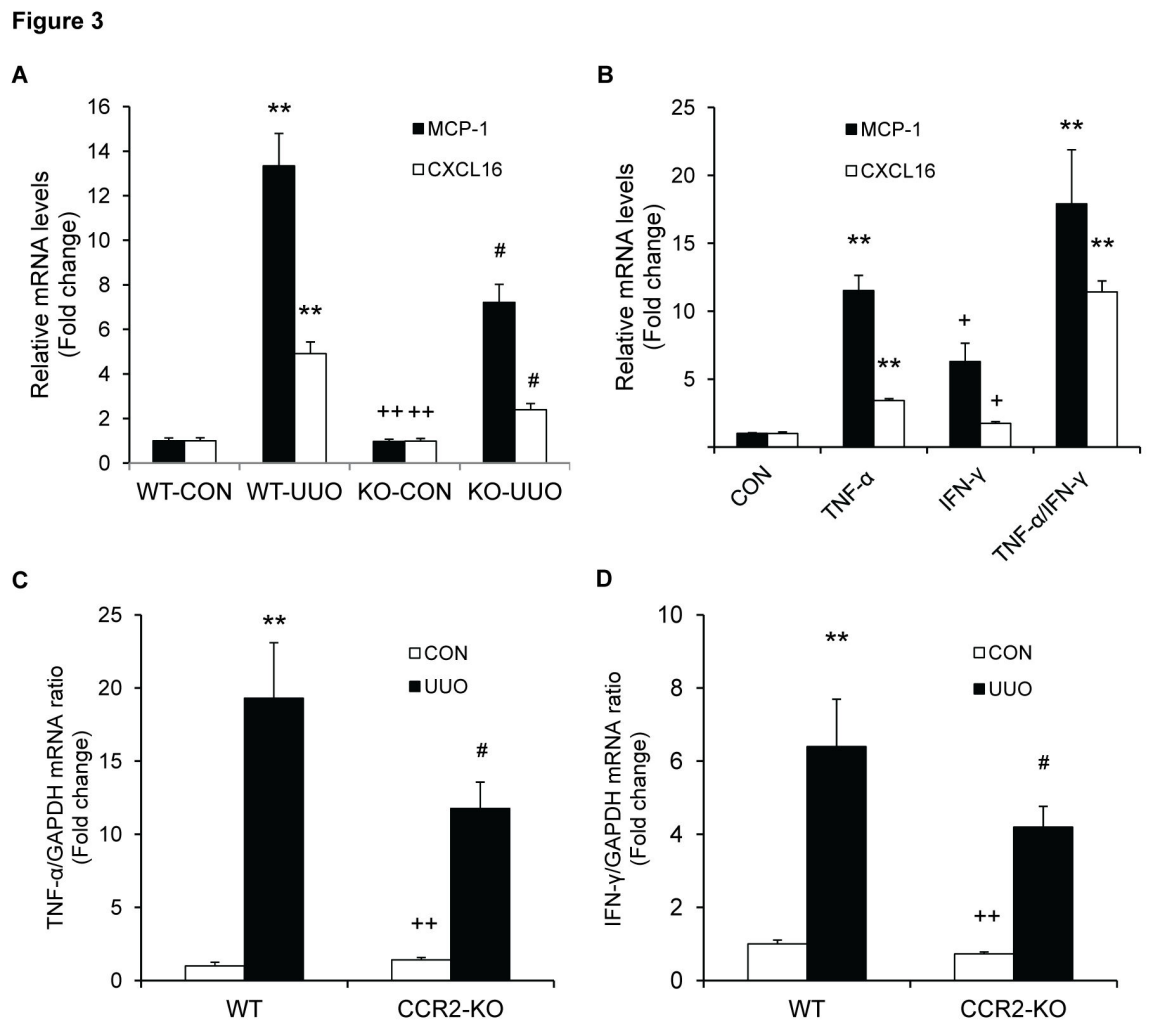
CCR2 deficiency reduces profibrotic chemokine production. A. CCR2 deficiency inhibits MCP-1 and CXCL16 gene expression in the kidneys 7 days after UUO. The mRNA levels of MCP-1 and CXCL16 in the kidneys of WT and CCR2-KO mice are determined by real-time RT-PCR. ** P < 0.01 vs WT controls, # P < 0.05 vs WT UUO and ++ P < 0.01 vs KO UUO. n=4 per group. B. TNF-α and IFN-γ synergistically up-regulate MCP-1 and CXCL16 mRNA expression in mouse tubular epithelial cells. The mRNA levels of CXCL16 in mouse tubular epithelial cells are determined by real-time RT-PCR. ** P < 0.01 vs vehicle controls. n=5 per groups. C. CCR2 deficiency inhibits TNF-α gene expression in the kidney 7 days after UUO. The mRNA levels of TNF-α in the kidneys of WT and CCR2-KO mice are determined by real-time RT-PCR. ** P < 0.01 vs WT controls, # P < 0.05 vs WT UUO and ++ P < 0.01 vs KO UUO. n=5 per group. D. CCR2 deficiency inhibits IFN-γ gene expression in the kidneys 7 days after UUO. The mRNA levels of IFN-γ in the kidneys of WT and CCR2-KO mice are determined by real-time RT-PCR. ** P < 0.01 vs WT controls, # P < 0.05 vs WT UUO and ++ P < 0.01 vs KO UUO. n=5 per group.

 We have recently demonstrated that the presence and development of bone marrow-derived fibroblasts from a CD45^+^ mononuclear cell population is driven by the induction of the chemokines - MCP-1 and CXCL16 [10,25]. We therefore examined if CCR2 deficiency affects MCP-1 and CXCL16 gene expression. Real time RT-PCR showed that genetic disruption of CCR2 inhibited MCP-1 and CXCL16 mRNA in the kidney in response to obstructive injury (Figure 3A). These data indicate that CCR2 signaling regulates chemokines – MCP-1 and CXCL16 gene expression. 

Since inflammatory cytokines induce chemokine gene expression [26], we investigated if TNF-α and IFN-γ can regulate the gene expression of MCP-1 and CXCL16 in mouse tubular epithelial cells. Our results showed that TNF-α significantly induced MCP-1 and CXCL16 gene expression, while INF-γ had only minor effect. Interestingly, TNF-α and IFN-γ synergistically induced MCP-1 and CXCL16 gene expression (Figure 3B). These results indicated that these inflammatory cytokines up-regulate MCP-1 and CXCL16 gene expression in mouse tubular epithelial cells. This prompted us to examine if CCR2 deficiency affected inflammatory cytokine expression. Our results showed that genetic disruption of CCR2 significantly reduced gene expression of TNF-α and IFN-γ in the kidney following obstructive injury (Figure 3C-D), which was associated with a reduction in macrophage infiltration into the kidneys in response to obstructive injury (Figure S2). 

### CCR2 Deficiency Inhibits Bone Marrow-derived Myofibroblast Formation

**Figure 4:**
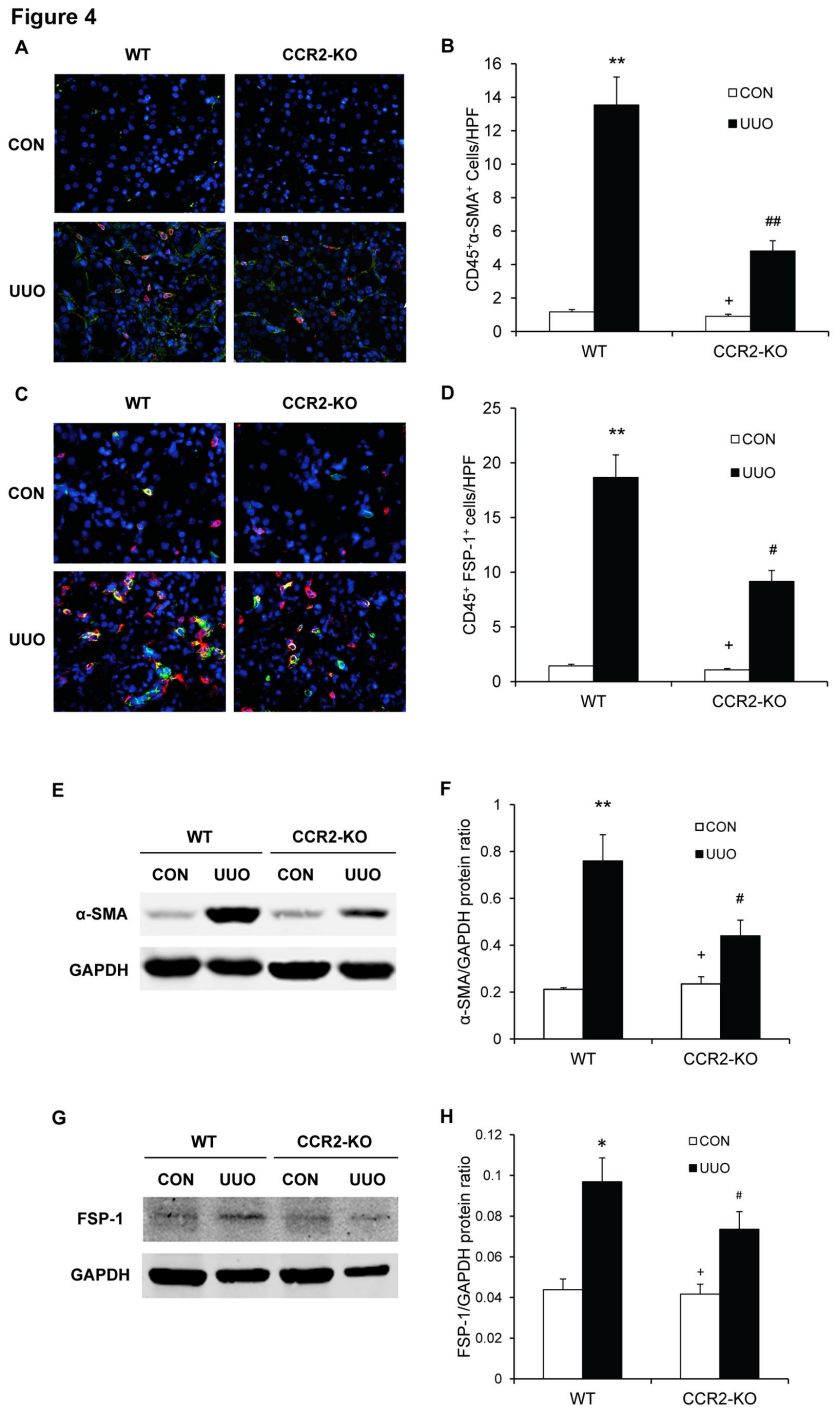
CCR2 deficiency inhibits bone marrow-derived myofibroblast formation in obstructive nephropathy. **A**. Representative photomicrographs of CD45 and α-SMA immunostaining in the kidneys of WT and CCR-KO mice 2 weeks after UUO. **B**. Quantitative measurements of α-SMA protein expression in the kidneys of WT and CCR2-KO mice 4 weeks after UUO. ** *P* <0.01 vs WT controls; ^##^ *P* <0.01 vs WT UUO; ^+^ *P* <0.05 vs KO UUO. n=6 per group. **C**. Representative photomicrographs of CD45 and FSP-1 immunostaining in the kidneys of WT and CCR-KO mice 2 weeks after UUO. **D**. Quantitative measurements of FSP-1 protein expression in the kidneys of WT and CCR2-KO mice 2 weeks after UUO. ** *P* <0.01 vs WT controls; ^#^ *P* <0.05 vs WT UUO; ^+^ *P* <0.05 vs KO UUO. n=6 per group. **E**. Representative Western blots show the levels of α-SMA protein expression in the kidneys of WT and CCR2-KO mice. **F**. Quantitative analysis of α-SMA protein expression in the kidneys of WT and CCR2-KO mice. ** *P* <0.01 vs WT controls; ^#^ *P* <0.05 vs WT UUO; ^+^ *P* <0.05 vs KO UUO. n=5 per group. **G**. Representative Western blots show the levels of FSP-1 protein expression in the kidneys of WT and CCR2-KO mice. **H**. Quantitative analysis of FSP-1 protein expression in the kidneys of WT and CCR2-KO mice. * *P* <0.05 vs WT controls; ^#^ *P* <0.05 vs WT UUO; ^+^ *P* <0.05 vs KO UUO. n=5 per group.

To determine if CCR2 deficiency influences bone marrow-derived myofibroblast transformation in the kidney, WT and CCR2 KO mice were subjected to UUO for 7 days. Kidney sections were stained for CD45 and α-SMA, a marker of myofibroblasts or CD45 and fibroblast-specific protein 1 (FSP-1), a marker for activated fibroblasts, and examined with a fluorescence microscope. The results revealed that genetic deletion of CCR2 resulted in a significant reduction in the number of CD45 and α-SMA, or CD45 and FSP-1 dual positive cells in obstructed kidneys compared with WT mice (Figure 4 A-D). Consistent with these findings, Western blot analysis showed that CCR2 deficiency significantly reduced the protein expression levels of α-SMA and FSP-1 in obstructed kidneys compared with WT mice (Figure 4 E-H). These results indicate that CCR2 deficiency reduces bone marrow-derived myofibroblasts formation. 

### CCR2 Deficiency Suppresses Renal Fibrosis

**Figure 5:**
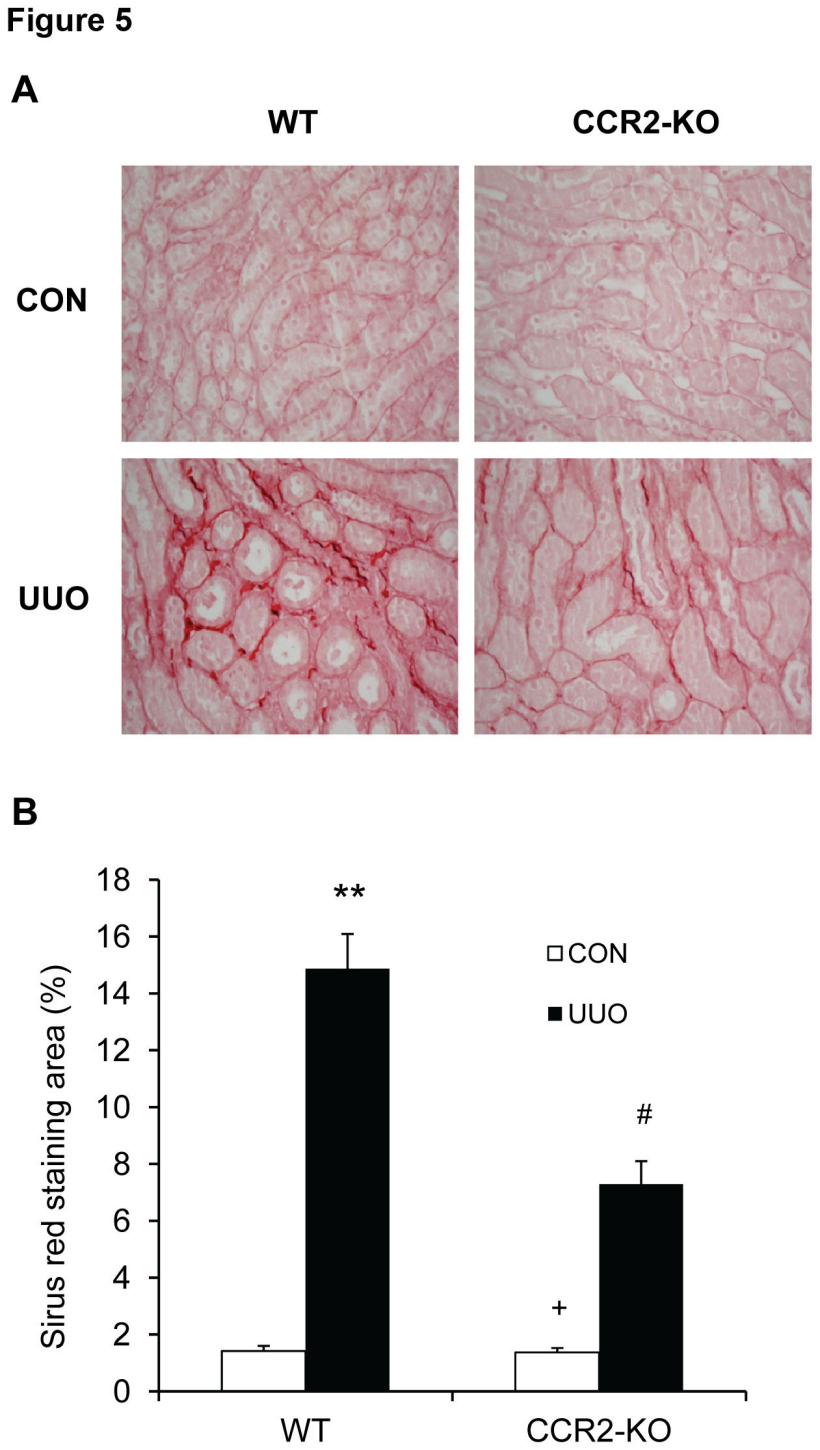
CCR2 deficiency suppresses renal fibrosis and extracellular matrix deposition in the kidney. **A**. Representative photomicrographs show kidney sections stained with picrosirius red for assessment of total collagen deposition. **B**. Bar graph shows quantitative analysis of renal interstitial collagen in different groups as indicated. ** P < 0.01 vs WT controls, + P < 0.05 vs KO UUO, and # P < 0.05 vs WT UUO. n= 6 per group.

Since CCR2 regulates the accumulation and activation of bone marrow-derived fibroblasts in the kidney in response to obstructive injury, we then examined the effect of CCR2 deficiency on the development of renal fibrosis. WT and CCR2 KO mice were subjected to UUO for 14 days. WT mice developed significant collagen deposition in obstructed kidneys as demonstrated by picrosirius red staining, whereas these responses were significantly attenuated in obstructed kidneys of CCR2 KO mice (Figure 5). These data indicate that CCR2 plays a critical role in the pathogenesis of renal fibrosis.

### CCR2 Deficiency Inhibits ECM Protein Expression

**Figure 6:**
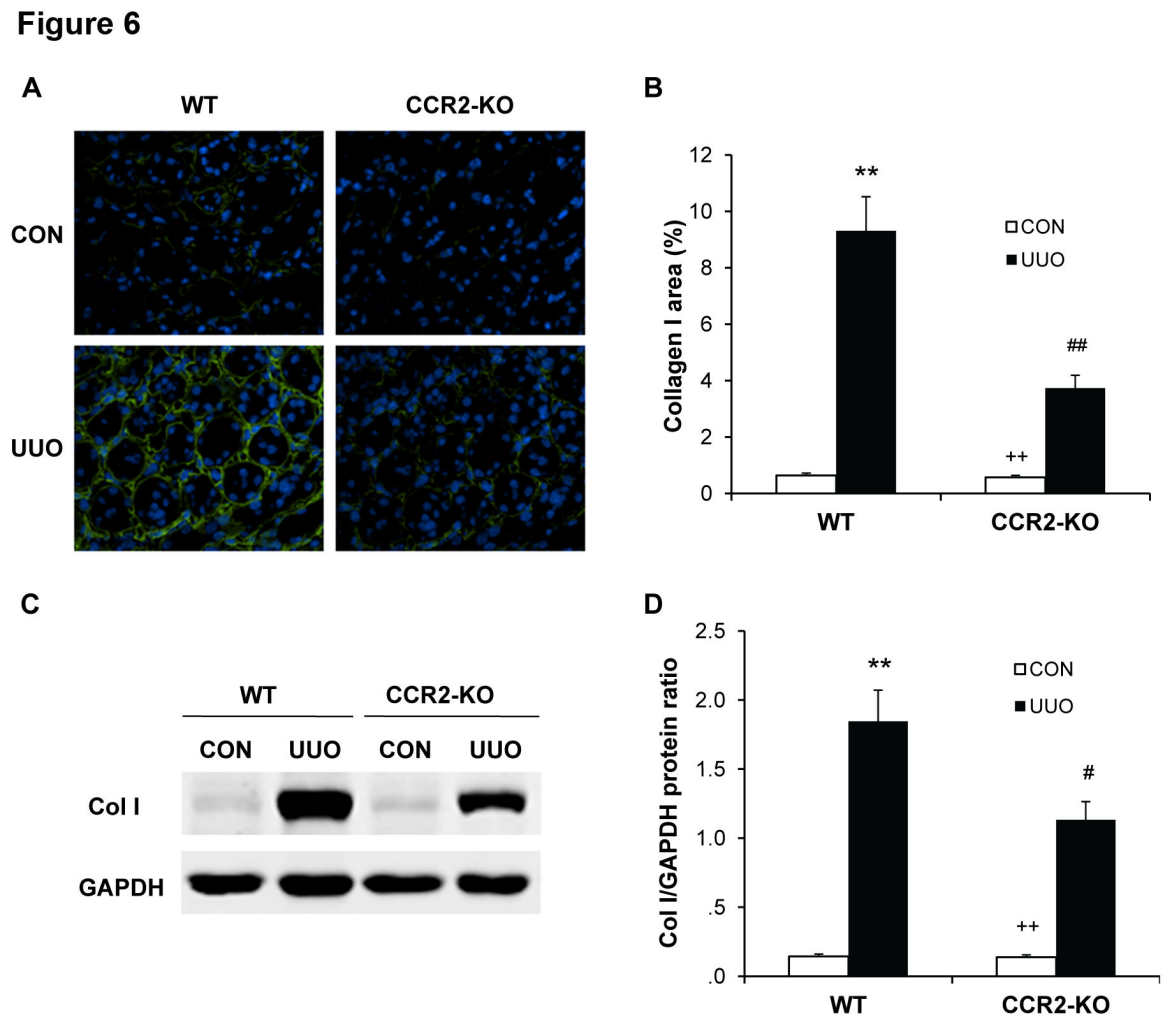
CCR2 deficiency inhibits collagen I expression in the kidney. **A**. Representative photomicrographs of collagen I immunostaining in the kidneys of WT and CCR2-KO mice at day 14 after surgery (original magnification X400). **B**. Quantitative analysis of interstitial collagen I protein expression in the kidney sections of WT and CCR2-KO mice. ** *P* < 0.01 vs WT-controls and ^##^ *P* < 0.01 vs WT UUO, and ^++^ *P* < 0.01 vs KO UUO. n=6 per group. **C**. Representative Western blots show the protein levels of collagen I in the kidneys of WT and CCR2-KO mice. **D**. Quantitative analysis of collagen I protein expression in the kidneys of WT and CCR2-KO mice. ** *P* < 0.01 vs WT controls, ^#^ *P* < 0.05 vs WT UUO, and ^++^ *P* < 0.01 vs KO UUO. n=5 per group.

**Figure 7:**
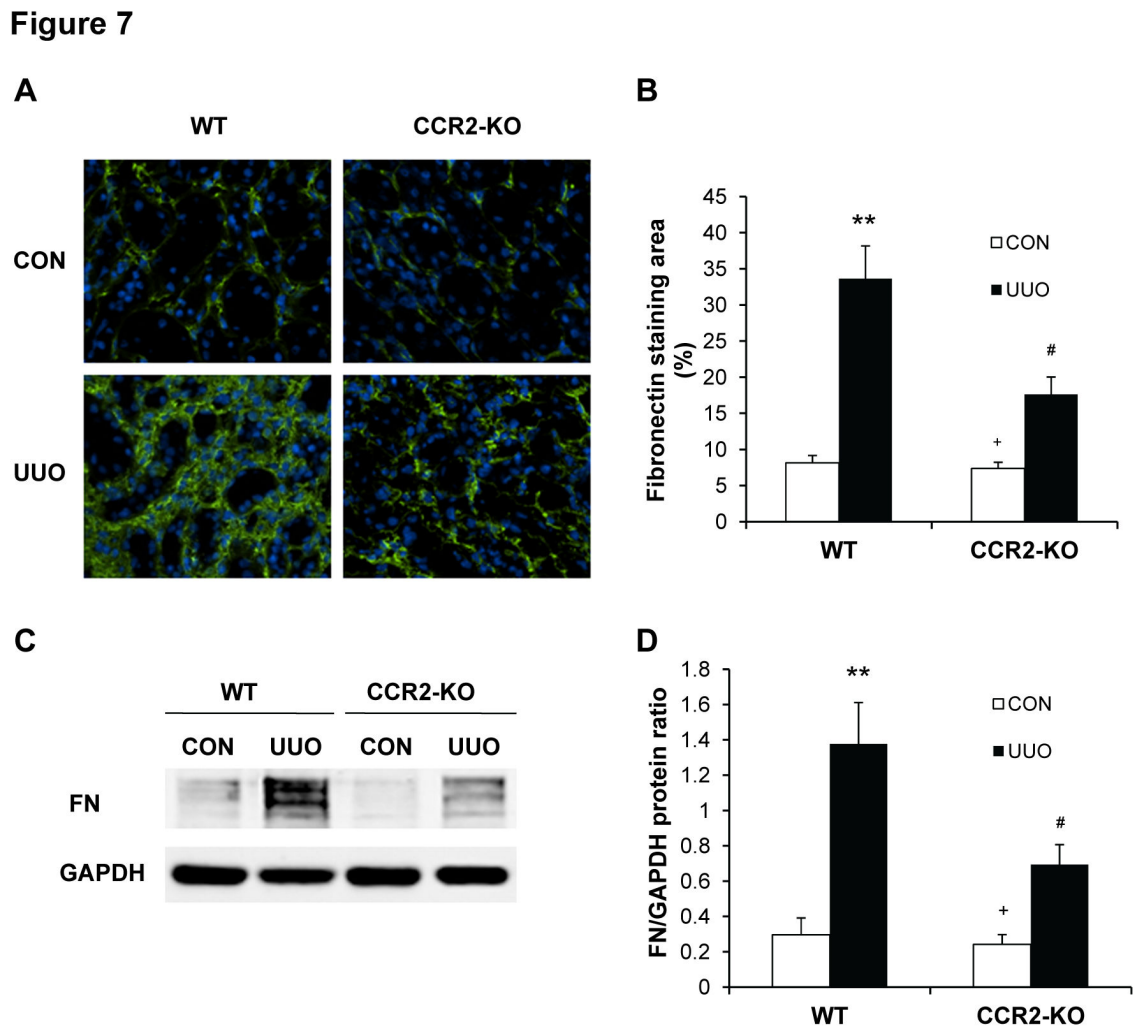
CCR2 deficiency reduces fibronectin expression in the kidney. **A**. Representative photomicrographs of fibronectin immunostaining in the kidneys of WT and CCR2-KO mice at day 14 after UUO (original magnification X400). **B**. Quantitative analysis of interstitial fibronectin protein expression in the kidney sections of WT and CCR2-KO mice. ** *P* < 0.01 vs WT controls, ^#^ *P* < 0.05 vs WT UUO, and ^+^ *P* < 0.05 vs KO UUO. n=6 per group. **C**. Representative Western blots show the protein levels of fibronectin in the kidneys of WT and CCR2-KO mice. **D**. Quantitative analysis of fibronectin protein expression in the kidneys of WT and CCR2-KO mice. ** *P* < 0.01 vs WT controls, ^#^ *P* < 0.05 vs WT UUO, and ^+^ *P* < 0.05 vs KO UUO. n=5 per group.

 We next investigated the effect of genetic deletion of CCR2 on the expression and accumulation of collagen I and fibronectin, two major components of ECM. There was a marked increase in the protein expression levels of collagen I and fibronectin in obstructed kidneys of WT mice, whereas genetic deletion of CCR2 significantly suppressed the protein expression levels of these matrix proteins in obstructed kidneys (Figure 6 and 7). These data indicate that genetic deletion of CCR2 attenuates renal fibrosis by inhibiting production and deposition of ECM proteins.

## Discussion

In this study, we demonstrate that (1) Bone marrow-derived fibroblasts express CCR2; (2) CCR2 deficiency inhibits bone marrow-derived fibroblast accumulation and macrophage infiltration in the kidneys in response to obstructive injury; (3) CCR2 deficiency inhibits MCP-1 and CXCL16 gene expression; (4) CCR2 deficiency suppresses the transformation of bone marrow-derived myofibroblast and the expression of α-SMA and FSP-1; (5) CCR2 deficiency reduces renal fibrosis and the expression of ECM proteins. These results indicate that CCR2 plays an important role in the development of renal fibrosis through recruiting bone marrow-derived fibroblasts and macrophages into the kidney.

Renal fibrosis is a hallmark of chronic kidney disease regardless of underlying etiologies. Activated fibroblasts are responsible for the excessive production of extracellular matrix. However, the origin of the fibroblasts that are responsible for the excessive production of extracellular matrix is still the subject of intense investigation. They are traditionally believed to arise from resident interstitial fibroblasts [27-29]. Recent studies indicate they may originate from epithelial/endothelial transition[30-33], and bone marrow-derived fibroblasts [7-11,30].

Bone marrow-derived fibroblast precursors termed fibrocytes are derived from a subpopulation of circulating mononuclear cells [12,17,18,34,35]. These cells express hematopoietic markers such as CD45 and CD11b and mesenchymal markers such as collagen I and vimentin. We and others have shown that these cells migrate into the kidney in response to obstructive injury and contribute to the development of renal fibrosis [7,10,13]. Furthermore, we have recently provided unequivocal evidence that bone marrow-derived fibroblasts migrate into the kidney in response to obstructive injury using bone marrow transplantation [10]. The donor mice express GFP under the control of collagen α1(I) promoter [23]. In the present study, we have shown that GFP^+^ cells express CCR2 and constitute 35-40% of PDGFR-β^+^ fibroblasts following obstructive injury.

Bone marrow-derived fibroblast express certain chemokine receptors such as CCR2, CCR5, CCR7, CXCR6, and CXCR4 in different organs and conditions [10,18,19,22]. This is important because chemokines through interaction with their receptors recruit these cells into area of injury [7,10,36,37]. In our previous study, we have shown that CXCL16 is induced in response to obstructive injury and plays an important role in recruiting bone marrow-derived fibroblasts into kidney and the development of renal fibrosis in a murine model of chronic kidney disease induced by unilateral ureteral obstruction [10]. CCR2 is a receptor for monocyte chemoattractant proteins (MCPs). In the present study, we have demonstrated that bone marrow-derived fibroblasts in the obstructed kidney express CCR2 and genetic disruption of CCR2 suppresses the accumulation of bone marrow-derived fibroblasts expressing CD45 and procollagen I or PDGFR-β and attenuates the degree of renal fibrosis. These data indicate that CCR2 is an important mediator for the recruitment of bone marrow-derived fibroblasts into the kidney in response to obstructive injury. This effect is likely related to MCP-1 induction in the kidney because we have shown that genetic deletion of MCP-1 suppresses the uptake of bone marrow-derived fibroblasts and the development of fibrosis in the heart [25]. 

We have recently shown that CXCL16 is induced in tubular epithelial cells in response to obstructive injury and targeted disruption of CXCL16 inhibits the recruitment of bone marrow-derived fibroblasts into the kidney and the development of renal fibrosis [10]. We have also shown that MCP-1/CCR2 axis plays an important role in the recruitment of bone marrow-derived fibroblasts into the heart during the development of cardiac fibrosis [22,25,38]. We therefore hypothesized that these chemokines and their receptors might be interactive. In support of this hypothesis, our results demonstrate that CCR2 deficiency inhibits MCP-1 and CXCL16 gene expression in the kidney in response to obstructive injury. Furthermore, our results reveals that MCP-1 and CXCL16 gene expression in tubular epithelial cells is induced by inflammatory cytokines - TNF-α and IFN-γ. CCR2 deficiency suppresses TNF-α and IFN-γ gene expression and monocyte/macrophage infiltration. These data indicate that MCP-1 mediated mononuclear cell infiltration may be the source of these inflammatory cytokines. Further study is needed to dissect the mechanisms underlying the induction of TNF-α and IFN-γ in the kidney. Nevertheless, our present study suggests for the first time that the interaction of two distinct chemokine systems modulates renal tubular epithelial cell-initiated fibrosis.

Myofibroblasts are a population of contractile fibroblasts that play an important role in wound healing and organ fibrosis [29]. It is generally thought that myofibroblasts are the main cells responsible for excessive ECM production during the development of kidney fibrosis [4,39]. However, their origin has been of intense debate. Using genetic tracing study, Lebleu et al. have recently shown that myofibroblasts arise from resident fibroblasts (50%), bone marrow-derived fibroblasts (35%), endothelial-to-mesenchymal transition (10%), and epithelial-to-mesenchymal transition (5%)[20]. Our present study demonstrates that bone marrow-derived myofibroblasts identified as CD45 and α-SMA dual positive cells accumulate in the kidneys of WT mice following obstructive injury, and their accumulation is significantly reduced in the obstructed kidneys of CCR2-KO mice. These results strongly indicate that CCR2 signaling play an important role in the development of bone marrow-derived myofibroblasts in the kidneys, which contribute to the population of renal myofibroblasts. 

A pathological feature of renal fibrosis is a striking increased production and deposition of extracellular matrix proteins such as collagens and fibronectin. Morphometric analysis of picrosirius red staining of kidney sections at day 14 after obstructive injury reveals increased interstitial collagen deposition. This fibrotic response is significantly attenuated in the obstructed kidneys of CCR2 KO mice. Consistent with these findings, we further demonstrate that the protein levels of collagen I and fibronectin are markedly increased in the injured kidneys of WT mice, whereas these responses are significantly reduced in the injured kidneys of CCR2 KO mice. These data indicate that CCR2 signaling regulates extracellular matrix protein production.

In summary, these data define a novel mechanism by which CCR2 participates in renal fibrosis. In response to obstructive injury, the activated CCR2 signaling contributes to recruit bone marrow-derived fibroblasts and macrophages into the kidney leading to the development of renal fibrosis. These data suggest that inhibition of CCR2 signaling could constitute a novel therapeutic approach for fibrotic kidney disease.

## Supporting Information

Figure S1 **CCR2 deficiency suppresses the accumulation of bone marrow-derived fibroblasts in the kidneys after UUO.** **A**. Representative photomicrographs of kidney sections from WT and CCR2-KO mice 5 days after UUO stained for CD45 (red), PDGFR-β (green), and counterstained with DAPI (blue). **B**. Quantitative analysis of CD45^+^ and PDGFR-β^+^ fibroblasts in the kidneys of WT and CCR2-KO mice 5 days after UUO. ** *P* < 0.01 vs WT controls, ^#^ *P* < 0.05 vs WT-UUO, and ^+^ *P* < 0.05 vs KO-UUO. n=6 per group.
(TIF)

Figure S2 **CCR2 deficiency reduces macrophage infiltration into the kidney after UUO.** **A**. Representative photomicrographs of kidney sections from WT and CCR2-KO mice 7 days after UUO stained for F4/80 (brown) and counterstained with hematoxylin (blue). **B**. Quantitative analysis of F4/80^+^ macrophages in the kidneys of WT and CCR2-KO mice 7 days after UUO. ** *P* < 0.01 vs WT controls, ^#^ *P* < 0.05 vs WT-UUO, and ^+^ *P* < 0.05 vs KO-UUO. n=6 per group.
(TIF)
