# Supplementary figures and images for: CCR2 Regulates the Uptake of Bone Marrow-Derived Fibroblasts in Renal Fibrosis
Source: PLoS One. 2013 Oct 10;8(10):e77493. doi: 10.1371/journal.pone.0077493 (PMC3795063; doi:10.1371/journal.pone.0077493)

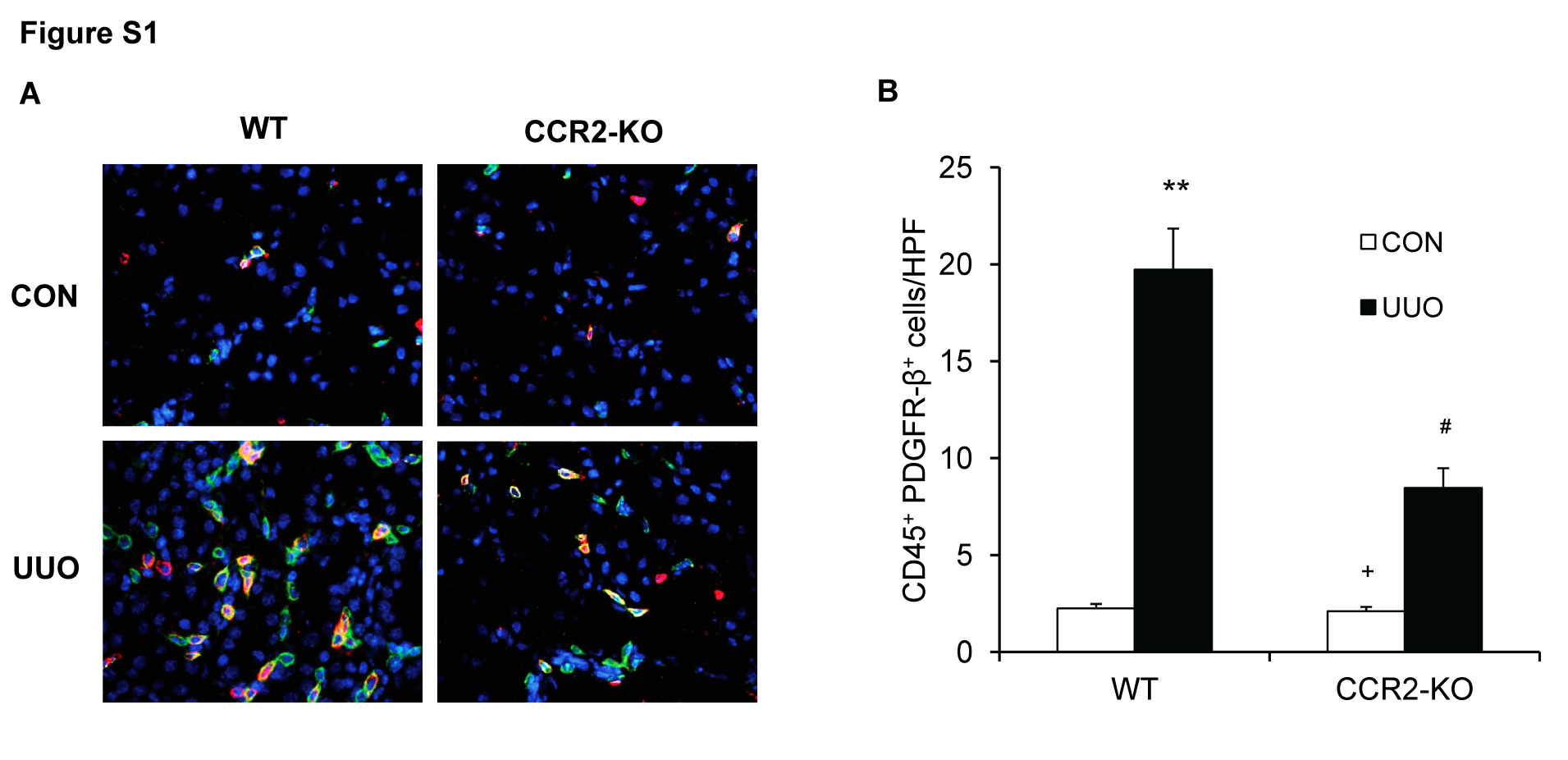

Supplement: Figure S1 — CCR2 deficiency suppresses the accumulation of bone marrow-derived fibroblasts in the kidneys after UUO. A. Representative photomicrographs of kidney sections from WT and CCR2-KO mice 5 days after UUO stained for CD45 (red), PDGFR-β (green), and counterstained with DAPI (blue). B. Quantitative analysis of CD45+ and PDGFR-β+ fibroblasts in the kidneys of WT and CCR2-KO mice 5 days after UUO. ** P < 0.01 vs WT controls, # P < 0.05 vs WT-UUO, and + P < 0.05 vs KO-UUO. n=6 per group. (TIF) [file pone.0077493.s001.tif]

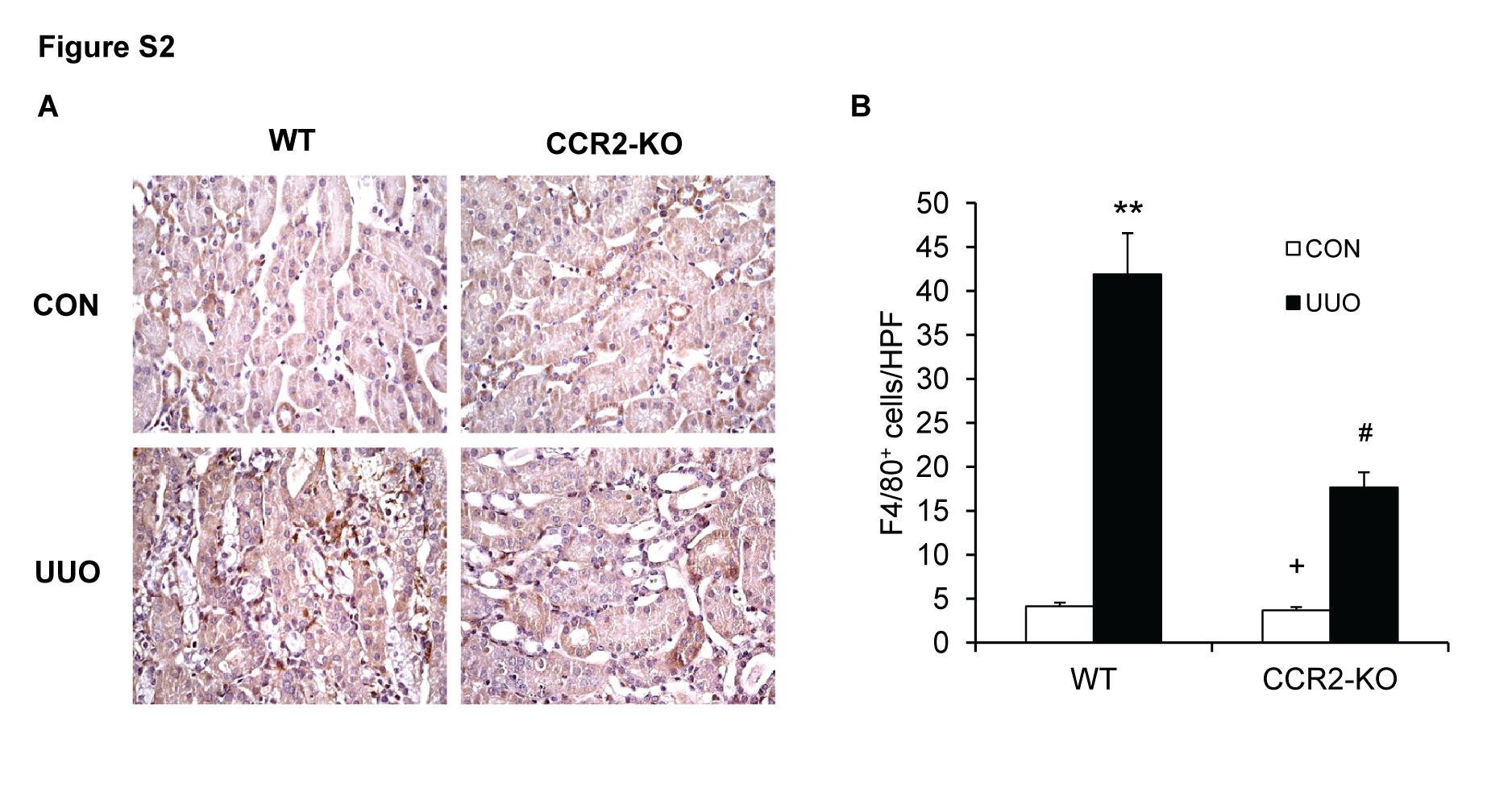

Supplement: Figure S2 — CCR2 deficiency reduces macrophage infiltration into the kidney after UUO. A. Representative photomicrographs of kidney sections from WT and CCR2-KO mice 7 days after UUO stained for F4/80 (brown) and counterstained with hematoxylin (blue). B. Quantitative analysis of F4/80+ macrophages in the kidneys of WT and CCR2-KO mice 7 days after UUO. ** P < 0.01 vs WT controls, # P < 0.05 vs WT-UUO, and + P < 0.05 vs KO-UUO. n=6 per group. (TIF) [file pone.0077493.s002.tif]
